# Inhibition of prenylated KRAS in a lipid environment

**DOI:** 10.1371/journal.pone.0174706

**Published:** 2017-04-06

**Authors:** Johanna M. Jansen, Charles Wartchow, Wolfgang Jahnke, Susan Fong, Tiffany Tsang, Keith Pfister, Tatiana Zavorotinskaya, Dirksen Bussiere, Jan Marie Cheng, Kenneth Crawford, Yumin Dai, Jeffrey Dove, Eric Fang, Yun Feng, Jean-Michel Florent, John Fuller, Alvar D. Gossert, Mohammad Hekmat-Nejad, Chrystèle Henry, Julia Klopp, William P. Lenahan, Andreas Lingel, Sylvia Ma, Arndt Meyer, Yuji Mishina, Jamie Narberes, Gwynn Pardee, Savithri Ramurthy, Sebastien Rieffel, Darrin Stuart, Sharadha Subramanian, Laura Tandeske, Stephania Widger, Armin Widmer, Aurelie Winterhalter, Isabel Zaror, Stephen Hardy

**Affiliations:** 1 Department of Global Discovery Chemistry, Novartis Institutes for BioMedical Research, Emeryville, California, United States of America; 2 Center for Proteomic Chemistry, Novartis Institutes for BioMedical Research, Basel, Switzerland; 3 Department of Oncology, Novartis Institutes for BioMedical Research, Emeryville, California, United States of America; 4 Department of Oncology, Novartis Institutes for BioMedical Research, Cambridge, Massachusetts, United States of America; Beatson Institute for Cancer Research, UNITED KINGDOM

## Abstract

RAS mutations lead to a constitutively active oncogenic protein that signals through multiple effector pathways. In this chemical biology study, we describe a novel coupled biochemical assay that measures activation of the effector BRAF by prenylated KRAS^G12V^ in a lipid-dependent manner. Using this assay, we discovered compounds that block biochemical and cellular functions of KRAS^G12V^ with low single-digit micromolar potency. We characterized the structural basis for inhibition using NMR methods and showed that the compounds stabilized the inactive conformation of KRAS^G12V^. Determination of the biophysical affinity of binding using biolayer interferometry demonstrated that the potency of inhibition matches the affinity of binding only when KRAS is in its native state, namely post-translationally modified and in a lipid environment. The assays we describe here provide a first-time alignment across biochemical, biophysical, and cellular KRAS assays through incorporation of key physiological factors regulating RAS biology, namely a negatively charged lipid environment and prenylation, into the *in vitro* assays. These assays and the ligands we discovered are valuable tools for further study of KRAS inhibition and drug discovery.

## Introduction

RAS proteins function as molecular switches to regulate cell growth, differentiation, and apoptosis through interactions with several effectors leading to multiple pathways emanating from this critical node in the cell [1, 2]. RAS bound to GTP is active/on, whereas RAS bound to GDP is inactive/off. Conversion between on/off states is regulated by guanine nucleotide exchange factors (GEFs) and GTPase-activating proteins (GAPs). RAS activity requires plasma membrane association and it has been shown that post-translational modification is important for membrane targeting and crucial for biological function [3–6]. There are three *ras* genes (*HRAS*, *NRAS*, and *KRAS*) that encode for 4 RAS proteins, because *KRAS* encodes 2 splice variants. The 4 proteins (HRAS, NRAS, KRAS4A and KRAS4B) are highly homologous in the sequences of their catalytic G-domain but differences exist in the *C*-terminal region, named the “hypervariable region” or HVR, which results in different post-translational modifications [7]. All 4 proteins undergo farnesylation (C15 isoprenoid), guided by a carboxyterminal CAAX motif which is subsequently methylated. HRAS, NRAS, and KRAS4A have an additional palmitoylation on a cysteine close to the *C*-terminus whereas KRAS4B has a poly-Lysine sequence close to the *C*-terminus, associating with negatively charged lipid head groups in the membrane. Recently, prenylated KRAS has been investigated by NMR in a lipid environment, clearly corroborating the importance of the membrane for RAS function [8].

It has been known since the 1980’s that RAS mutations are the driver in about 30% of cancers with high prevalence in carcinomas of the pancreas, colon, and lung [9, 10]. Mutations (mostly codons 12, 13 and 61) lead to constitutively active RAS through impairment of intrinsic and GAP-stimulated GTP hydrolysis activity, leading to persistent formation of RAS-GTP. Constitutively active RAS drives unregulated cell division, promotes and maintains tumor angiogenesis, and drives seeding of metastases. For the past 3 decades, many strategies have been devised and executed to pharmacologically target mutant-RAS tumors, including strategies to inhibit RAS directly, to interfere with correct localization of RAS, or to inhibit effector pathways downstream of RAS [10–12]. None of these approaches have been able to progress to an effective cancer therapy. Most of the recent efforts have focused on inhibiting RAS directly, either through covalent approaches [13–15] or non-covalent approaches targeting allosteric sites or protein-protein interaction surfaces [16–21]. These approaches were all driven by assays using truncated non-prenylated forms of RAS, including biophysical binding assays, nucleotide exchange assays (which target the GDP-bound form of RAS), or assays measuring the disruption of a protein-protein interaction. A correlation between the various *in vitro* assays and cellular activity has been challenging since none of these assays assess the biological switching function of RAS. In addition, a correlation between cellular activity and biophysical binding affinity has been elusive [19].

Our interest is in inhibition of effector activation through small molecules that interact directly with GTP-loaded mutant-RAS. Inspiration for our approach originally came from published ^31^P-NMR studies showing that GTP-HRAS exists in an equilibrium of two conformations where one state is selected by effector binding, state 2(T), and the other state is prominent in mutants unable to bind effectors, state 1(T) [22–24]. In addition, RAS has been shown to be highly dynamic and various analyses have pointed at transient pockets that could be accessible for ligand interaction [25–29]. From this, we hypothesized that small molecules could interact with GTP-bound RAS in a transient druggable pocket resulting in stabilization of a conformation that is unable to activate effectors. Further support for this hypothesis came from the work describing compounds that stabilize the GTP-RAS 1(T) conformation [24, 30, 31].

**Fig 1:**
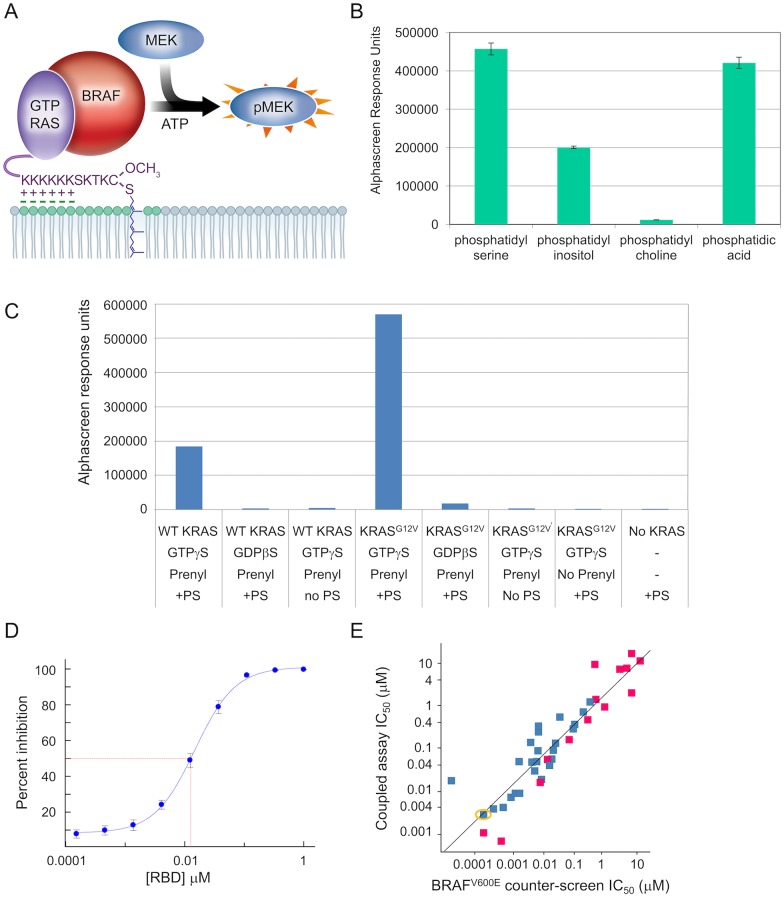
Coupled KRAS-BRAF-MEK assay recapitulates known KRAS biology. (A) Schematic of the assay components: GTP-loaded full-length, prenylated KRAS^G12V^ (purple); full-length BRAF (red); biotinylated MEK1^K97R^ (blue); phosphatidylserine (negatively-charged lipid head-groups in green). Readout happens at the pMEK level using alphascreen technology with streptavidin donor beads, protein A acceptor beads, and an anti-phospho-MEK antibody. (B) AlphaScreen response units measuring phospho-MEK levels resulting from KRAS activation of BRAF in the presence of different phospholipids; the KRAS preparation is wildtype, prenylated protein, loaded with GTPγS. (C) AlphaScreen response units measuring phospho-MEK levels resulting from KRAS activation of BRAF, assessing dependence of the coupled assay on nucleotide, prenylation, PS, and KRAS mutation status (WT = wildtype); nucleotides are GDPβS and GTPγS, non-hydrolyzable analogues of GDP and GTP respectively. (D) Dose-response curve for titrating the RAS-Binding domain of CRAF in a coupled assay with GTPγS-loaded, prenylated KRAS^G12V^ in the presence of PS; IC_50_ = 0.013 μM ± 0.002 μM (geomean ± standard deviation, N = 4) (E) Correlation plot of IC_50_ values in the BRAF^V600E^ counter-screen *vs* IC_50_ values in the coupled assay (with GTPγS-loaded, prenylated KRAS^G12V^ in the presence of PS) for a diverse subset of RAF inhibitors from historical programs, including (in yellow circle) compound C from reference [33]; magenta are Type-I inhibitors and blue are Type-II inhibitors. R^2^ of the regression line is 0.83.

Since we wanted to measure inhibition of effector activation, we designed a coupled pathway assay, measuring KRAS4B activation of BRAF kinase, which phosphorylates inactive MEK (Fig 1A). We focused on mutant KRAS4B (referred to as KRAS in the remainder of this paper), which is the KRAS splice variant that is prevalent in cancer and has been shown to require the *C*-terminal farnesyl group, the *C*-terminal poly-Lysine block and a membrane/lipid environment with negatively charged head-groups for biological function [3, 4, 32]. A counter-screen to the coupled assay removed kinase inhibitors, resulting in the identification of KRAS-specific inhibitors. Cellular assays confirmed functional effects and the structural basis for inhibition was assessed by NMR. Direct binding of inhibitors to KRAS was determined by biosensor-based binding studies which demonstrated that the potency of inhibition in the biochemical and cellular assays matched the affinity of binding only when KRAS is in its native state, namely post-translationally modified and in a lipid environment. Evidence of the direct binding of the inhibitors to KRAS in these biosensor-based studies, as well as the alignment between the *K*_d_s and the potencies in the biochemical and cellular assays, support the hypothesis that these assays are modulated through the direct interaction of the inhibitors with KRAS.

## Results

### Protein preparation for assay development

Several preparations of purified KRAS were created to compare wildtype *vs* mutant and prenylated *vs* non-prenylated proteins. The prenylated KRAS proteins were expressed in insect cells using a baculovirus system and produced as a mixture of farnesylated and geranyl-geranylated forms in a roughly 40:60 ratio (based on mass spectrometry analysis). The purity of the various KRAS preparations was ≥ 95%. The preparation of BRAF was also produced in insect cells and was partially purified to ~ 50%. This preparation could be activated by KRAS (characteristics described below) and was co-purified with endogenous insect cell 14-3-3, which is a known RAF co-factor [34, 35]. Any further purification resulted in loss of ability to be activated by KRAS. Finally, in order to avoid MEK inhibitors from interfering with the coupled assay read-out, we used a preparation of MEK^K97R^, an inactive form of that protein.

### Biochemical assay measures inhibition of KRAS

At the time of assay development for the coupled assay, no small molecule inhibitors of RAS had been disclosed so we relied on recapitulating the known biology for assay validation. As a first step, we examined phospholipid dependencies as shown in Fig 1B. This experiment confirms observations reported by others that KRAS activation of BRAF requires a negatively charged lipid environment, while a neutral lipid such as phosphatidylcholine was unable to support KRAS activation of BRAF [32]; for further assay development we chose phosphatidylserine (PS). Fig 1C shows that KRAS activation of wildtype BRAF is dependent on the presence of protein prenylation, PS, and the presence of a GTP analogue (GTPγS, which hydrolyzes at a lower rate than GTP). Low level basal activity of the wildtype BRAF was increased 50-fold or more with the addition of prenylated, GTP-loaded KRAS along with PS, which was prepared as a solution of liposomes and added to assay buffer. When using KRAS loaded with a non-hydrolyzable GDP analogue (GDPβS) or when omitting PS or prenylation, only the basal level activity of BRAF was observed. Comparing wildtype (WT) KRAS with KRAS^G12V^ showed that both proteins are able to activate BRAF, with the mutant achieving a higher level of activation. All further coupled assay experiments utilize GTPγS-loaded, prenylated KRAS^G12V^ in a PS environment.

As a control to assess inhibition of the KRAS activation of BRAF, we added the RAS-Binding-Domain (RBD) of CRAF and showed that this blocked the activation with an IC_50_ of 0.013 μM (Fig 1D). RAF inhibitors are also expected to inhibit in this coupled assay (due to inhibition of the kinase activity of BRAF) so we designed a counter-screen that matches the coupled assay but is lacking KRAS. As is shown in Fig 1C, the wildtype BRAF preparation that can be activated by KRAS is not able to phosphorylate MEK without KRAS so we used BRAF^V600E^ to drive the phosphorylation of MEK in the counter-screen. We tested a diverse set of RAF inhibitors from historical programs in both the coupled assay and the counter-screen and showed that the coupled assay IC_50_s correlated well with the IC_50_s in the BRAF^V600E^ counter-screen across five orders of magnitude (Fig 1E). The results from the control experiments with RBD and with RAF inhibitors provide reassurance that the BRAF preparation used in the coupled assay behaves as expected.

### Validated hits inhibit KRAS function

**Fig 2:**
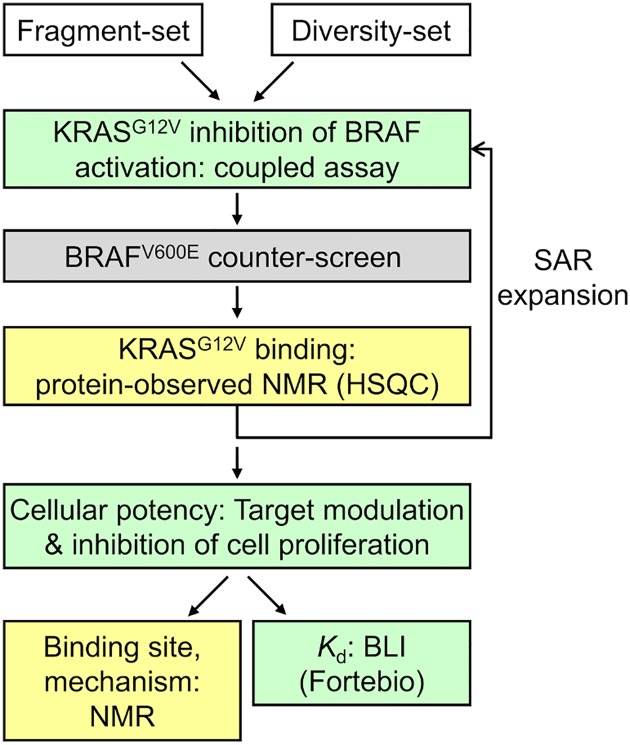
Workflow to identify and validate chemical matter that inhibits KRAS activation of BRAF through direct interaction with KRAS. Green boxes utilize full-length prenylated KRAS in a lipid environment, yellow boxes utilize truncated KRAS in aqueous buffer and the grey box is the BRAF^V600E^ counter-screen.

We identified KRAS-specific inhibitors following the workflow in Fig 2, which includes the KRAS^G12V^-dependent coupled assay with the BRAF^V600E^ counter-screen, hit-validation by protein-observed NMR, determination of cellular potency, and biophysical assessments. With respect to the KRAS-dependent coupled assay, 8800 diverse fragments (average molecular weight 251) were screened at 250 μM and 8800 diverse small molecules (average molecular weight 349) were screened at 50 μM. We defined primary hits as those compounds with ≥ 80% inhibition in the coupled assay and ≤ 20% inhibition recorded from historical BRAF^V600E^ screening efforts. The hit-rate after this selection step was 1.9%. In parallel, a fragment library of 500 diverse fragments was screened by NMR against KRAS^G12V^. From this NMR-based fragment screen, we identified indoles as a privileged KRAS binding scaffold. After hit-list triaging and hit assessment from the coupled assay screen, we selected 17 compounds (3 containing the indole substructure) for hit confirmation in dose-response format for the coupled assay and counter-screen as well as hit validation by NMR.

**Fig 3:**
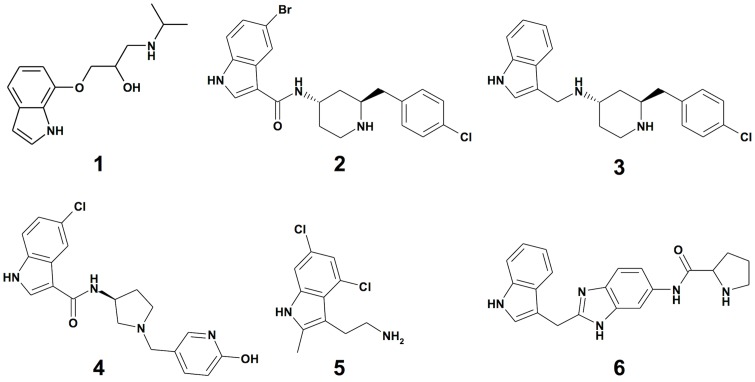
Chemical structures of compounds 1–6.

The first validation of these hits was focused on confirming a direct interaction with KRAS^G12V^ for which we used a protein-observed NMR Heteronuclear Single-Quantum Correlation (HSQC) assay. This assay was performed using non-prenylated GDP-KRAS^G12V^ since we found a lack of important resonances in KRAS bound to a GTP analogue due to dynamic conformational exchange. Two indole-containing fragments scored positive in the HSQC assay and also confirmed in the dose-response assays, including compound **1** (Fig 3) which recorded a coupled assay IC_50_ of 87 μM and an IC_50_ in the counter-screen of >250 μM (Table 1).

**Table 1:** Summary of biochemical, cellular, and biophysical measures of potency in μM.

	Compound 1	Compound 2	Compound 3	Compound 4	Compound 5	Compound 6
Coupled assay IC_50_^1^	87±22 (N = 8)	0.8±0.7 (N = 18)	1.2 (1.8;0.8)	49 (>25;49)	7 (7.0;7.0)	133 (>50;133)
Counter-screen IC_50_^1^	>250 (N = 11)	85±47 (N = 3)	>25 (>25;>25)	115 (>25;115)	29 (30;29)	134 (>50;134)
Growth inhibition EC_50_^2^	>30	2.7±1.4 (N = 3)	1.2	>30	18	>30
*K*_d_ BLI^3^	ND	0.8	2.6±0.8 (N = 10)	>10	34	1,100
*K*_d_ HSQC	ND	690 (603;776)^4^	ND	ND	1,100^5^	340^6^

Data for the coupled assay and for the growth inhibition assay, as well as the *K*_d_ from BLI reflect experiments with full-length prenylated GTPγS -loaded KRAS in a lipid environment. The *K*_d_ from HSQC reflects experiments with non-processed GDP-loaded KRAS in aqueous buffer. The counter-screen is the BRAF^V600E^ counter-screen. ND = Not Determined.

^1^IC_50_ values are shown as the geomean where at least two non-qualified values were available, with individual IC_50_ values shown in parentheses for every compound done with N = 2 and geomean ± standard deviation shown for compounds **1** and **2**.

^2^EC_50_ values are shown from curve-fitting where each concentration was run in triplicate and all points were used for fitting a single curve; EC_50_ value for compound **2** is shown as the geomean ± standard deviation from three independent assays.

^3^BLI measurements were performed in singleton after establishing reproducibility for compound **3**, which is shown as geomean ± standard deviation.

^4^Average value calculated from the shifts obtained from fitting the data related to two separate peaks (individual values noted in parentheses).

^5^Reference [19].

^6^Reference [18].

We explored the SAR around compound **1** by substructure and similarity searches from the existing compound archive as well as focused medicinal chemistry efforts, resulting in several compounds with improved potency in the coupled assay and similar binding behavior to the KRAS^G12V^ protein by NMR. Subsequent experiments focused on two active analogues from this effort (compounds **2** and **3**, Fig 3), as well as three control compounds. Structures are shown in Fig 3 for all compounds and their IC_50_ values from the coupled assay and counter-screen are shown in Table 1. The active analogues **2** and **3** had IC_50_ values in the coupled assay of 0.8 μM and 1.2 μM, respectively, with a good window of selectivity in the RAF-based counter-screen, where minimal activity was observed. Compound **4** has negligible biochemical activity in the coupled assay, yet similar physical chemical properties compared to compounds **2** and **3**. Compounds **5** and **6** are RAS ligands identified by others [18, 19]. Compound **5** exhibits a 7 μM IC_50_ in the coupled assay and a small selectivity window in the counter-screen; compound **6** has negligible activity in both the coupled assay and the counter-screen.

**Fig 4:**
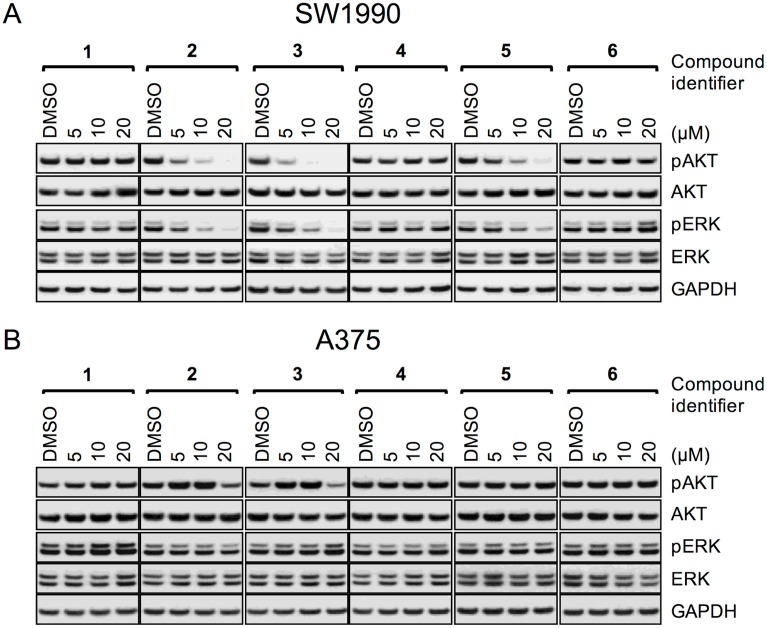
Cellular activity of ligands on signaling in KRAS and BRAF mutant tumor cells. Dose-response data for all compounds from Fig 3 on SW1990 cells (A) and A375 cells (B), assessing impact on pAKT and pERK, together with their total protein controls and a GAPDH control.

Cellular effects of the compounds were assessed by Western blot analysis and inhibition of cell proliferation. Fig 4A shows the dose-dependent effects of the compounds on pAKT and pERK, representing two different pathways downstream of KRAS^G12D^ in SW1990, a human pancreatic cancer cell line. Inhibition of cell proliferation was recorded as an EC_50_ value for the compounds in SW1990 cells (Table 1). The active analogs **2** and **3** showed single digit μM potency in these cellular assays, with dose-dependent decreases in phosphorylation of both of the pathway markers, while total protein levels were not affected, consistent with on-target inhibition of KRAS^G12D^. The negative control compound **4** and the initial fragment hit compound **1** were inactive at the concentrations tested, in line with their activities in the coupled assay. As published previously, compound **5** showed double-digit μM potency in cells and compound **6** was inactive in cells [18, 19]. All compounds tested show good correlation between their IC_50_ in the coupled assay measuring inhibition of the KRAS switching function and their cellular EC_50_. As a critical control for non-specific inhibition of RAS, we tested the compounds in a melanoma cell line driven by BRAF^V600E^ (A375) and pERK levels were not affected by the compounds (Fig 4B). The pAKT levels in this cell line show an unexpected pattern of modulation for the active analogs **2** and **3**, which is consistent with on-target RAS activity since the PI3K-AKT pathway is still expected to be regulated by RAS in these cells.

### NMR studies with truncated KRAS protein

**Fig 5:**
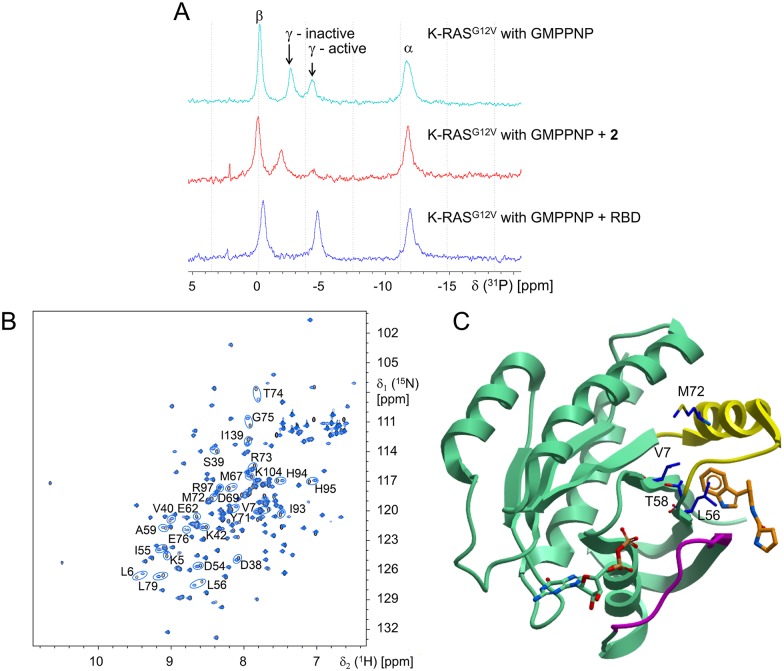
NMR experiments probe mechanism and binding site. (A) ^31^P-NMR spectrum of non-prenylated GMPPNP-loaded KRAS^G12V^ shows two environments for the γ-phosphate (top) with a shift to a single inactive population with 1 mM compound **2** (middle) and a single active population with 0.5 mM of the CRAF-RBD (bottom); α and β phosphate have single environments and the KRAS^G12V^ is present at 5 mg/ml. (B) ^15^N,^1^H-HSQC spectrum for non-prenylated GDP-KRAS^G12V^ in absence (black) or presence (blue) of 400 μM compound **2** (protein concentration is 0.5 mg/ml). The residues with major chemical shift changes are indicated with circles. (C) Residues engaged in nOe’s between non-prenylated GDP-KRAS^G12V^ and compound **2** shown in blue stick model with labels using the crystal structure for compound **6** (PDB-ID 4EPY) as reference; compound **6** in orange, GDP and RAS protein in green, switch-I in magenta and switch-II in yellow.

In order to assess mechanism of inhibition and establish the binding site for the new inhibitors, we employed NMR studies using compound **2** and non-prenylated KRAS^G12V^ in the absence of PS. It has been previously reported that GTP RAS has two conformational states that can be detected by ^31^P-NMR [36, 37]. These states are seen in splitting the γ-PO_4_ resonance peak into two peaks. Each peak has been associated with either an inactive conformation or an active conformation by recording spectra from inactive HRAS (T35S) or by trapping HRAS or KRAS in its active form with RBD and comparing those spectra with the two-state spectrum from GTP RAS. We have repeated these experiments using non-prenylated KRAS^G12V^ loaded with the non-hydrolyzable GTP analogue GMPPNP and compound **2** (Fig 5A). The relationship of the ^31^P γ-PO_4_ peaks for KRAS^G12V^ and KRAS^G12V^ plus RBD are as described previously [36]. We find that compound **2** traps KRAS in a conformation lacking the γ-^31^P resonance of the “active” RBD binding form, whereas the γ-^31^P resonance corresponding to the “inactive” form persists and is slightly shifted further downfield, comparable to the observation of the inactive HRAS mutant.

Next, we assessed the KRAS binding site for these compounds. The HSQC NMR shifts for compound **2** observed in non-prenylated KRAS^G12V^ are shown in Fig 5B. Many of the residues showing significant shifts belong to a conformationally flexible region around the switch-II loop, known to be involved in effector activation. Further NMR experiments resulted in assignment of multiple intermolecular nOe’s between indole protons of compound **2** and V7, L56, T58, and M72 of non-prenylated KRAS^G12V^, confirming a direct and specific interaction. The binding site indicated by these residues has since been disclosed by others using similar truncated protein preparations and different ligands [18–20]. Fig 5C maps the residues involved in the intermolecular nOe’s between compound **2** and KRAS onto the public domain crystal structure of one of these published co-crystal structures, namely KRAS and compound **6** (PDB-ID 4EPY). This mapping shows that the indole from compound **2** binds at the same site as the indole from compound **6**, which is also the same site as the indole from compound **5** (as observed in PDB-ID 4DST).

An HSQC titration experiment for compound **2** with non-prenylated KRAS^G12V^ showed an estimated *K*_d_ of 690 μM (Table 1), well above the IC_50_ from the coupled assay (0.8 μM). Since ligands in this series show cellular effects in the low micromolar range (Fig 4A and Table 1), there is a significant decrease in potency when comparing the affinity in the biochemical and cellular assays with the affinity determined in the NMR experiment. A similar disconnection between cellular activity and binding affinity was reported for compound **5** [19]. We hypothesize that this difference is due to the use of full-length prenylated KRAS in a native or native-like lipid environment in the cellular and biochemical assays, whereas the NMR assessments used truncated non-prenylated KRAS in aqueous buffer lacking PS.

### Biolayer interferometry studies with prenylated KRAS protein

To further evaluate if the biochemical and cellular potencies of the compounds are consistent with binding to KRAS, and to test the hypothesis that the affinity of binding to KRAS is dependent on the presence of the *C*-terminal prenyl group and the presence of lipids, we explored the binding of compounds directly to prenylated KRAS using ForteBio’s biolayer interferometry (BLI) platform [38]. In this method, KRAS is attached to a sensor surface via streptavidin:biotin interactions, and the binding of compounds to the target is measured directly. The *K*_d_s for the compounds determined for prenylated KRAS^G12V^ loaded with GTPγS in the presence of PS (prepared as a solution of liposomes) correlate well with their biochemical IC_50_s and the cellular EC_50_s (Table 1), and the rank-order of the compounds is consistent across the assays. Of specific note is that control compound **4** was inactive in the biochemical and cellular assays and did not bind in BLI studies at 10 μM. Compounds **5** and **6** also showed *K*_d_s in the BLI assay that correlate with their biochemical IC_50_ and cellular EC_50_ values (Table 1). These results support the hypothesis that the binding of these inhibitors to KRAS results in an effect in the coupled assay and in the cellular assays.

**Fig 6:**
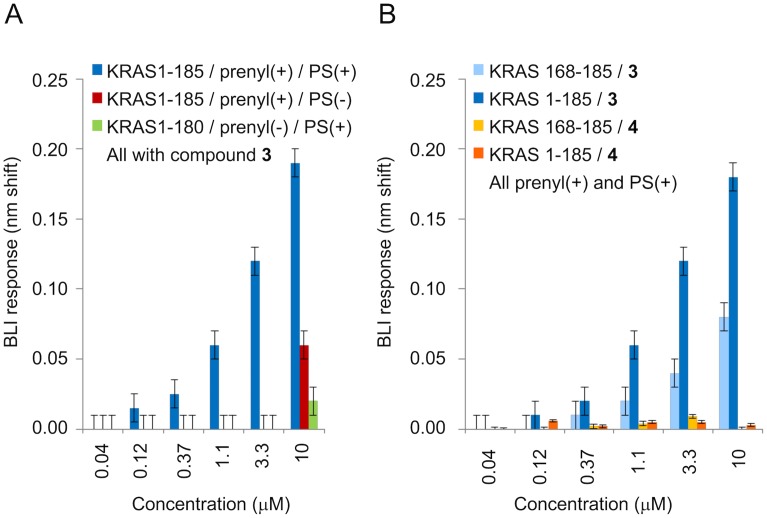
BLI experiments assess dependence of K_d_ on prenylation, PS, and HVR. (A) Response data at various concentrations of compound **3** with different protein preparations (with or without prenylation, with or without PS). Error bars represent assay method variability (three standard deviations for buffer). (B) Dose-response data for compounds **3** and **4** with prenylated HVR (light blue & yellow) and with full-length prenylated KRAS^G12V^ (dark blue & orange); all in PS. Error bars represent assay method variability (three standard deviations for buffer).

To understand the impact of prenylation and the presence of PS, we performed a series of control experiments with BLI. First, a positive control was selected to ensure run-to-run reproducibility so that results could be compared across studies. Compound **3** was selected because it showed good behavior up to 10 μM, whereas compound **2** showed minor non-specific binding at 10 μM (*K*_d_ determinations described earlier were not impacted). In the absence of PS or for KRAS constructs lacking the *C*-terminus (and thus the prenyl group), binding for compound **3** was significantly lower. Specifically, minimal binding was observed in a KRAS construct 1–180, which lacks the *C*-terminus (and therefore the prenyl group), at 10 μM of compound **3**. For full-length KRAS, but in the absence of PS, no binding was observed up to 3.3 μM of compound **3**, and some binding was observed at 10 μM. The differences in responses for these controls and full-length KRAS in the presence of PS are shown in Fig 6A. We also observed that none of the compounds bind non-specifically to biosensors lacking KRAS. These observations show that the correlation between affinity in the BLI binding assay and potency in the coupled biochemical assay is dependent on full-length prenylated KRAS and PS.

Because we compared KRAS constructs with and without the prenylated *C*-terminus, we also examined the binding of our inhibitors to an 18-residue KRAS *C*-terminal prenylated peptide. This peptide is immobilized to the biosensor through biotin and has a farnesyl group attached to the cysteine residue through the thioether link: Biotin-GGG GGG GGE KMS KDG KKK KKK SKT KC (-OCH3)-Farnesyl. Surprisingly, we observed that compound **3** binds to the prenylated peptide in a concentration-dependent manner in BLI experiments with PS (Fig 6B). The response profiles for compound **3** binding to the peptide were more complex than those for compound **3** binding to KRAS protein, showing possible non-specific interactions with the peptide. In this control experiment, binding signals for the compound do not fully dissociate to background levels at 3.3–10 μM, and some secondary binding is observed in the association phase. Consistent with KRAS binding studies using the full-length protein, control compound **4** did not bind to the prenylated peptide (Fig 6B).

## Discussion

Therapeutically relevant inhibitors of mutant KRAS should block the activation of the multiple downstream effector pathways. This premise led us to the design of a novel KRAS-BRAF-MEK coupled biochemical assay which recapitulates the known biology of the KRAS switch and allows for an assessment of inhibition of the KRAS switching function by small molecules. The use of the BRAF^V600E^ counter-screen and of inactive MEK allowed us to eliminate confounding kinase inhibitors from our hit finding efforts. We utilized these biochemical assays in a hit-finding campaign to identify KRAS-specific inhibitors that were shown to be active in KRAS-driven cells. The biosensor-based method BLI was used to show that the compounds interact directly with KRAS. The suite of assays we developed focused on key physiological factors regulating RAS biology, namely phosphatidylserine and prenylation. This enabled for the first time a good correlation between potencies determined in a biochemical assay assessing the KRAS switching function, a biophysical assay using full-length prenylated KRAS, and cellular assays assessing RAS-driven target modulation and inhibition of cell proliferation.

The coupled assay monitors the effector activation function of KRAS on BRAF and was shown to be dependent on the full-length prenylated protein and the presence of the negatively charged lipid PS. These findings are consistent with observations of the critical role of the farnesylated *C*-terminal hypervariable region (HVR) and of the negatively charged phospholipids (PS in particular) on KRAS. For example, detailed biophysical and computational experiments of KRAS localization, KRAS orientation on the membrane, and KRAS regulation of spatial cycling through subcellular compartments, paint a picture of intricate regulation and importance of the full-length prenylated protein [8, 39–43]. Binding of prenylated KRAS to nanodisc, and subsequent binding of the CRAF-RBD to KRAS, was shown to be dependent on PS [44] and interfering with the PS content of the plasma membrane was shown to mislocalize KRAS and impact signaling [45, 46]. The dependencies of KRAS activity on the presence of GTP nucleotide, prenylation, and PS (Fig 1C) is consistent with proper functioning of the KRAS switch in this coupled assay. Furthermore, the agreement between biochemical, cellular, and biophysical potency measurements of inhibitors identified using this coupled assay indicates this assay is relevant as a model for cellular potency and can therefore be used in hit-finding campaigns aimed at identifying small molecule KRAS inhibitors.

It should be noted that the BRAF preparation is not a pure protein preparation. The molecular details of RAS activation of BRAF are currently not fully understood (reviewed in [47]) and based on the observation that further purification of the BRAF fraction resulted in loss of ability to be activated, it seems that additional proteins or co-factors could play a role. KRAS purity, in contrast, was ≥ 95% and the good correlation between the biochemical IC_50_s in the KRAS-BRAF coupled assay and the biophysical *K*_d_s obtained with KRAS in the absence of BRAF strongly suggests that the observed inhibition in the coupled assay is driven by the direct binding of both novel and previously published inhibitors to KRAS. Fig 1C also shows that the mutant form of the KRAS protein achieves a higher level of activation compared to wildtype KRAS. We speculate that this may be related to the fact that mutant KRAS was shown to be released from the occluded auto-inhibited state compared to wildtype KRAS in nanodisc NMR experiments for KRAS^G12D^ [8] and KRAS^G12V^ (M. Ikura, personal communication).

The chemical matter discovered through the hit-finding and hit-validation efforts driven by the coupled assay is exemplified by compounds **2** and **3**. These compounds exhibit the expected profile in a cellular context where we show modulation with low micromolar potency of two pathways emanating from RAS in the SW1990 KRAS^G12D^ driven cell line. The compounds have no effect on the pERK levels in cells that are driven by BRAF^V600E^ which is constitutively active and independent of RAS [48], supporting the conclusion that the pERK effects seen in SW1990 are RAS-dependent. The pattern of pAKT modulation by our active analogs in A375 (BRAF^V600E^) cells cannot be fully explained at this time, however, RAS is still expected to be an activator of the PI3K-AKT pathway and RAS effectors have been shown to be regulated by allosteric mechanisms which could lead to paradoxical effects [49].

Biophysical and structural work on RAS proteins has historically been dominated by the use of truncated protein preparations where the membrane anchors have been omitted. Our initial work on exploring the binding site and mechanism of our inhibitors was performed with similar truncated proteins and our nOe experiments highlighted a binding site (the “indole site”) that others have also discovered [18–20].

The ^31^P-NMR experiments with GTPγS-loaded KRAS link the binding of compound **2** to a mechanism that involves trapping mutant RAS in an inactive conformation. The binding affinity of the inhibitor based on HSQC NMR experiments using truncated GDP-KRAS was found to be three orders of magnitude weaker than the potency observed in the biochemical and cellular context where prenylated protein and a lipid environment are present. This disconnection prompted further biophysical studies to assess the role of the *C*-terminal membrane-targeting functionality in the mechanism of these inhibitors. Initial NMR experiments using prenylated protein in the presence of lipids showed interactions of compound **2** with the lipids. In addition, important HSQC NMR resonances in KRAS become invisible when bound to a GTP analogue due to dynamic conformational exchange. This makes HSQC NMR more difficult to use for the goal of correlating affinity with potency of inhibition, since the latter requires the GTP-bound form. These observations prompted the need for a different method for affinity determinations. A BLI-based biosensor assay was developed to measure direct binding of the compounds to KRAS. In these experiments we used the same liposome and protein preparations that were used in the coupled biochemical assay in order to assess whether a correlation exists between binding affinity and potency of inhibition.

The BLI experiments using full-length, prenylated GTPγS-loaded protein in a PS context align well with the potency observed in biochemical and cellular settings for the inhibitors disclosed here as well as two inhibitors published by others (Table 1), suggesting that all these assays are measuring interactions of the compounds with the same species of KRAS. Specifically, our inhibitors show inhibition with potency values in cellular and biochemical assays that are in the low micromolar range which match the *K*_d_s determined in the BLI experiments. Compound **5** was reported to have a potency of 16 μM in cell-based assays [19], which aligns with our IC_50_ and EC_50_ results and with the BLI *K*_d_ using prenylated protein and PS. Compound **6** has no reported cellular activity [18] which correlates with a coupled assay IC_50_ of > 250 μM and a BLI *K*_d_ of 1,100 μM. The published *K*_d_s for compounds **5** and **6** to truncated GDP-loaded KRAS in absence of a lipid environment were 1 mM and 340 μM respectively (Table 1; [18, 19]), highlighting also a disconnection for compound **5** when comparing biochemical and cellular potencies with binding affinity to truncated protein. All these findings support the notion that the inhibition observed in the coupled assay and in cellular assays for compounds **2**, **3** and **5** is dependent on the KRAS protein being in the biologically relevant form (prenylated) and context (membrane-like environment).

The control BLI experiments using the *C*-terminal prenylated peptide from KRAS as a binding partner indicate that compound **3** interacts with this peptide in the PS environment in a concentration range near the *K*_d_ observed for the binding to the full-length, prenylated protein. The experiment suggests that the interactions of the compound with the peptide may involve a mixture of specific and non-specific interactions. The interaction of cationic drugs with anionic liposomes has been reported previously [50] and we expect that some of the non-specific interactions we observe are driven by direct interaction of compounds with liposomes. The observation of the impact of PS on compound potency and the suggestion of compound binding to the *C*-terminal peptide evoke important questions. Further studies will be required to determine the mechanism of inhibition of these compounds which could involve a direct interaction with the membrane or with the HVR, and/or an effect of lipid anchoring on the conformation or oligomeric state of KRAS. It is interesting to note in this context that recently published models of membrane-associated prenylated KRAS place the “indole site” close to the lipid interface in at least one of the accessible conformational states [8, 29, 41, 42].

In summary, we developed a multi-component biochemical assay based on the KRAS-BRAF-MEK pathway that was used to identify cellularly active compounds that bind and inhibit KRAS. The biochemical assay is dependent on the presence of PS, and the presence of the lysine-rich *C*-terminus of KRAS, which is prenylated. Only when these conditions are fulfilled, do we find that the potency in both the biochemical and biophysical BLI assays is consistent with the potency observed in cell-based assays. 2D NMR studies show that the compounds bind in a known KRAS pocket distal to the GTP-binding site, and biophysical BLI analysis indicates that the inhibitors bind to KRAS, whereas a negative control compound does not. The alignment of potencies in the biochemical and cellular assays with the biophysical BLI *K*_d_s suggests that the mechanism of action involves the direct interaction of the inhibitors with KRAS. Determination of the mechanistic aspects of the presence of PS on KRAS activity is technically challenging, and warrants further study. Our work suggests that *in vitro* KRAS assays that more closely mimic the cellular context may provide more relevant inhibitors in KRAS-dependent cancer cells. The elucidation of the exact mechanism of action of our compounds in KRAS-dependent cancer cells will impact the search and characterization of inhibitors of KRAS and possibly other membrane-associated proteins.

## Material and methods

### Protein preparation

The KRAS constructs His-Tev KRAS^G12V^(1–180) and His-Tev Avi-Tag (Gly)8 KRAS^G12V^(1–180) were cloned in the pET22b E. coli plasmid (Novagen) with standard molecular biology techniques and the genes were driven by the T7 promoter. The BirA gene was cloned in a pACYC184 derived *E*. *coli* plasmid and is driven by the tac promoter. The plasmid with His-Tev KRAS^G12V^(1–180) was transformed into Rosetta2 DE3 *E*. *coli* cells (Novagen) and grown in a fermenter at 37°C to an OD600 = 8-10/ml in YEP medium using carbenicillin and chloramphenicol for selection, induced with 0.81mM IPTG and grown to OD600 = 30 at 18°C. The His-Tev Avi-Tag (Gly)8 KRAS^G12V^(1–180) and the BirA plasmids were co-transformed into BL21 (DE3) *E*. *coli* cells (Invitrogen) and grown in flasks in LB medium at 37°C using carbenicillin and chloramphenicol for selection. Induced at 1–2 OD/ml with IPTG to 0.5 mM and grown at 18°C to OD 4/ml. GST-CRAF RBD (aa51-131) construct was cloned in the *E*. *coli* pGEX2T plasmid (GE Healthcare) and is driven by the tac promoter. This plasmid was transformed into BL21 *E*. *coli* cells (Invitrogen) and grown in flasks in LB medium at 37°C using carbenicillin for selection. Induced at 1–2 OD/ml with IPTG to 0.5 mM and grown at 37°C for 4 hrs.

KRAS full length, BRAF full length and BRAF^V600E^ full length genes were cloned in the baculovirus expression vector pBlueBac4.5 (Thermo Fisher Scientific) and were driven by the polyhedrin promoter. These plasmids were subsequently used to generate baculovirus by the co-transfection/plaque purification method [51]. Viruses generated from the transfected insect cells were amplified using a standard low MOI infection method. Avi-tag-(Gly)8 –KRAS^G12V^ (2–188) was cloned into the pFastBac1 vector under the polyhedrin promoter. MEK1^K97R^-cAvi tag and BirA were cloned pFastBac Dual vector, driven by the polyhedrin and the p10 promoters respectively. The pFastBac1 plasmids containing Avi-tag-(Gly)8 –KRAS^G12V^ (2–188) and the pFastBac Dual vector with Avi-MEK^K97R^ and BirA were used to generate bacmid DNA as instructed in the Bac-to-Bac manual (Invitrogen). Baculoviruses expressing the recombinant proteins were generated using the Bac-To-Bac Expression System method (Invitrogen) following manufacturer’s protocol. The viruses generated from the transfected insect Sf9 cells were amplified using a standard low MOI infection method. The pFastBac1 plasmids containing Avi-tag-(Gly)8 –KRAS^G12V^ (2–188) and the pFastBac Dual vector with Avi-MEK^K97R^ and BirA were used to generate bacmid DNA as instructed in the Bac-to-Bac manual (Invitrogen). Baculoviruses expressing the recombinant proteins were generated using the Bac-To-Bac Expression System method (Invitrogen) following manufacturer’s protocol. The viruses generated from the transfected insect Sf9 cells were amplified using a standard low MOI infection method. To generate recombinant proteins for all the insect cell expressed proteins, suspension cultures of insect cells (Sf9, Sf21 or Tn5) were seeded at a density of 1.5x10^6^ cells/ml and infected with virus at an MOI of 10 or with a 3% volume. The infected insect cells were cultured for 48 hours at 27°C shaking at 120 rpm using 2L glass Erlenmeyer flasks and serum free media. Cells were harvested two days post-infection. To generate biotinylated Avi-tagged protein, a suspension culture of insect cells (Tn5) were seeded at a density of 1.5x10^6^ cells/ml and co-infected with virus at an MOI of 5:5 (recombinant baculovirus and BirA-expressing virus, both at passage 2). D-biotin was added to 50uM final concentration.

Cells were collected by centrifugation and resuspended in ~5x volumes of lysis buffers. *E*. *coli* cells were lysed by sonication or Microfluidizer (Microfluidics) and insect cells were lysed by Dounce homogenization. The proteins expressed in *E*. *coli* or baculovirus/insect cells were purified by a combination of affinity and size-exclusion chromatography.

More detailed descriptions of cloning, expression and purification are provided in S1 Protocols.

### Compound synthesis and characterization

Detailed descriptions of synthetic methods and compound characterization are provided in S2 Protocols.

### Phospholipid preparation for biochemical and biophysical assays

Phosphatidylserine (PS) liposomes were prepared by suspending the lipid (porcine brain; Avanti Polar Lipids #840032P) at 5 mg/ml (13 mM) in 50 mM Tris buffer, pH 7.5. The suspension was extruded 11 times through a 50 nm pore polycarbonate membrane using an Avestin LiposoFast-Basic Membrane Extruder and the solution was stored at 4°C. This method is similar to previously described methods for the preparation of phosphatidylcholine-based liposomes containing PS [52] with the exception that PS was the only lipid solubilized and it was dissolved directly in 50mM Tris pH 7.5 prior to extrusion. The resulting liposomes were characterized in triplicate by dynamic light scattering on a Wyatt DynaPro reader and were 120nm (polydispersity 16nm). The expected sizes for 100 and 300nm Nanosphere^™^ size standards (ThermoFisher Scientific 3100A and 3300A, respectively) were observed. Upon addition of KRAS, the liposomes were 129nm (polydispersity 19nm) in the presence of assay buffer.

### Assay for BRAF activation by KRAS

Reactions were run in 50 mM Tris, pH 7.0, 5 mM MgCl_2_, 0.01% BSA (Sigma #4503), 0.01% TritonX-100, 100 mM NaCl and 1 mM DTT. BRAF activation was measured as the amount of phosphorylation of inactive MEK protein. The AlphaScreen technology (PerkinElmer #6760671) with an anti-phospho-MEK antibody (Cell Signaling #9121) was used to detect MEK phosphorylation. Recombinant G12V-KRAS (2 nM final), either prenylated or nonprenylated, and either pre-loaded with GDPβS or GTPγS, was combined with PS (final 2 μg/ml), biotinylated MEK (final 20 nM) and ATP (final 10 μM) at 2x for 30 minutes prior to the addition of 2x full length wildtype BRAF fraction. The wildtype BRAF preparation was not quantified but used at a dilution that minimized basal activity. Reactions were stopped with the addition of EDTA plus Tween, final 20 mM and 0.003%, respectively. A mixture of AlphaScreen beads, composed of streptavidin donor and protein A acceptor beads, and anti-phospho-MEK antibody in 50 mM Tris, pH 7.5 and 0.01% Tween 20 was added for a final 20 μg/ml AlphaScreen beads and 1:2000 dilution of antibody. After an overnight incubation, the samples were read for emission at 570 nm after excitation of the donor beads at 680 nm on an Envision 2101 instrument (PerkinElmer). An initial survey of phospholipids was done with recombinant wildtype KRAS (final 100 nM), prenylated and pre- loaded with GPTγS, biotinylated MEK (final 40 nM), ATP (final 50 uM) and full length wildtype BRAF fraction. The liposomes phosphatidylinositol (Avanti Polar Lipids #840042P), L-α-phosphatidylcholine (Avanti Polar Lipids #840051P) and L-α-phosphatidic Acid (Avanti Polar Lipids #840101P) were prepared as described for PS liposomes above. All liposomes were assessed at 100 μg/ml.

For library screening and IC_50_ determinations (including the control experiment with RBD), the 2x mixture of RAS, lipid, MEK and ATP was combined with compound in DMSO at 2x for 30 minutes before the addition of 2x RAF to start the reactions. The final DMSO concentration was 2.5%. This assay was reproducible from run to run. The geomean of the IC_50_ ± the standard deviation for a known RAF inhibitor, compound C from [33], was 0.002±0.003 μM (N = 53). The geomean of the IC_50_ ± the standard deviation for compound **2** was 0.8±0.7 μM (N = 18). After establishing reproducibility with these compounds, all other compounds were measured with N≥2.

### Counter assay to eliminate BRAF inhibitors

An assay consisting of full length BRAF^V600E^ was used to flag inhibitors acting directly on BRAF, taking advantage of the constitutive activity of the BRAF mutant. The BRAF^V600E^ stock was diluted to give a reasonable signal upon phosphorylation of inactive MEK protein. The counter assay buffer, other components and order of addition matched the RAS-dependent assay except for the presence of KRAS. This assay was reproducible from run to run. The geomean of the IC_50_ ± the standard deviation for a known RAF inhibitor, compound C from [33], was 0.0007±0.0007 μM (N = 49). After establishing reproducibility with this compound, all other compounds were measured with N≥2.

### NMR experiments

All NMR experiments except for the ^31^P experiments were performed at 296K on a Bruker AV600 or AV800 NMR spectrometer, operating at 600 MHz or 800 MHz proton frequency, respectively, and equipped with cryoprobes. The buffer contained 20mM Tris HCl, pH8.0, 50mM NaCl, 1mM MgCl_2_, 2mM DTT. HSQC experiments were run with ^15^N-labeled and GDP-loaded K-RAS^G12V^ (1–169) at a concentration of 0.5 mg/ml, and ^13^C,^15^N-edited NOESY spectra were recorded with ^13^C,^15^N-labeled and GDP-loaded K-RAS^G12V^ (1–169) at a concentration of 7 mg/ml. The same samples were used to record triple-resonance experiments for resonance assignment. The NMR fragment screen was carried out using GDP-loaded KRAS^G12V^ (1–180). 500 fragments from a fragment library [53] were screened by T1ρ ligand observation experiments, followed by protein-observed HSQC experiments. ^31^P NMR spectra were acquired at 278°K on a Bruker AV400 spectrometer operating at 161.6 MHz ^31^P frequency, equipped with a BBO probe. Unlabeled GMPPNP-loaded K-RAS^G12V^ (1–169) was used at a concentration of 5 mg/ml alone or with 1 mM compound **2** or 0.5 mM of the CRAF-RBD.

NMR *K*_d_ values were determined by titrating ^15^N-labeled GDP-loaded K-RAS^G12V^ (1–169) protein with increasing amounts of compound **2** and following the resulting chemical shift perturbations. The weighted change in chemical shift of two peaks was calculated using a ^15^N scaling factor of 0.1 and the *K*_d_ calculated by fitting the data with an equation describing the Langmuir binding isotherm.

For binding site determination, 0.4 mM samples of KRAS^G12V^ (1–169) with intrinsically bound GDP were prepared in presence of 1 mM compound **2**. As reported earlier, backbone and methyl-group assignments were obtained with automated projection spectroscopy [54] and complete side-chain assignments with an H(C^ali^C^aro^)H-TOCSY [55]. For obtaining inter-molecular NOEs, double-half filter experiments were used [56, 57]. Additionally, a high-resolution spectrum was recorded on a specifically ILVM ^13^C-methyl labeled sample [58], in order to resolve ambiguities in NOE assignments.

### Cell lines and reagents

SW1990 and A375 cell lines were obtained from the Broad-Novartis Cancer Cell Line Encyclopedia collection and cultured in DMEM or RPMI supplemented with 10% FBS. Anti-p-ERK1/2 T202/Y204, -ERK1/2, -p-AKT S473, and -AKT antibodies were purchased from Cell Signaling. Anti-GAPDH was purchased from Millipore. MSD Phospho/Total ERK1/2 Whole Cell Lysate Kit (K15107D) was purchased from Meso Scale Discovery. CellTiter-Glo^®^ Luminescent Cell Viability Assay was purchased from Promega.

### Western blot analysis

Cells were seeded in 6-well plates and treated with compounds or DMSO at the indicated concentrations for 3hrs. Total cell lysates were prepared in either M-PER (Pierce) or RIPA lysis buffer (50 mM Tris-HCl, pH 8.0, 150 mM NaCl, 0.5% NP-40, 0.5% sodium deoxycholate) supplemented with 1x protease inhibitor cocktail (Sigma) and 1x phosphatase inhibitor cocktails (Sigma). Lysates were normalized for protein concentration, resolved by SDS-PAGE, transferred onto nitrocellulose membranes and probed with the indicated antibodies.

### Proliferation assay

Cells were seeded in 384-well plates and incubated with a 1:3 serial dilution of compound starting from 30 μM at 37°C with 5% CO_2_ for 5 days. Cell viability was measured using the CellTiter-Glo^®^ Luminescent Cell Viability Assay kit (Promega) and read on an Envision (Perkin Elmer) plate reader. Percent growth inhibition was calculated by normalizing treatment to DMSO control. EC_50_ values, where cell growth is inhibited by 50%, were calculated from dose response curves generated in GraphPad PRISM 6.07 using a non-linear regression 4-parameter curve fitting model. For every compound, each concentration was run in triplicate and all points were used for fitting a single curve. Compound **2** was tested in three independent assays and the geomean of the EC_50_ ± the standard deviation was 2.7±1.4 μM.

### Determination of binding constants with biolayer interferometry

Determination of *K*_d_s was performed on the Octet RED platform, according to the manufacturer's recommendations in their application note on small molecule analysis. The prenylated KRAS peptide containing the farnesyl group attached to the terminal cysteine (biotin-GGGGGGGGEKMSKDGKKKKKKSKTC(farnesyl)-OCH_3_) was obtained from AnaSpec (Fremont, California). Proteins were obtained as described above. The assay buffer was 25mM Tris containing 190mM glycine, 5mM MgCl_2_, 0.01% Triton X-100, 1mM DTT and 1.3uM PS (liposome preparation as described above). The protein was attached to SuperStreptavidin biosensors (ForteBio 18–5057) via Avi-tagged-KRAS G12V:GTPγS with *C*-terminal prenylgroup by adding 2μL of the 5mg/mL solution of PS to 2mL of assay buffer, followed by the KRAS protein to a final concentration of 40nM. After incubation for 1h at RT, the protein was loaded into a 96-well plate (Greiner 655209), and loaded a BLI response of 3-4nm. These sensors were subsequently blocked with a 100 μg/mL solution of biocytin, and a 6-point, 3X dilution series of compound was analyzed in the presence of 2.5% DMSO at 28°C. A reference sensor was run to remove systematic drift, and a second set of SuperStreptavidin biosensors blocked with biocytin was examined to remove systematic optical interferences, and to check for non-specific binding of compounds to the biosensor. *K*_d_s were determined using the ForteBio analysis software tool, which determines *K*_d_ from the steady-state plot, or from the determination of the kinetic constants *k*_on_ and *k*_off_. This assay was reproducible from run to run; the *K*_d_ for compound **3** obtained from the steady-state model with multiple preparations of PS liposomes was 2.6±0.8 μM (geomean ± standard deviation; N = 10). After establishing reproducibility with compound **3**, all other compounds were measured in singleton.

## Supporting information

S1 Protocols Detailed descriptions of cloning, expression and purification for protein preparation. (PDF)

S2 Protocols Detailed descriptions of synthetic methods and compound characterization. (PDF)
