# Supplementary material for: Inhibition of prenylated KRAS in a lipid environment
Source: PLoS One. 2017 Apr 6;12(4):e0174706. doi: 10.1371/journal.pone.0174706 (PMC5383040; doi:10.1371/journal.pone.0174706)
Supplement: S1 Protocols — (PDF) [file pone.0174706.s001.pdf]

Inhibition of Prenylated KRAS in a Lipid Environment
Supporting Information: S1 Protocols

***E. coli* expression constructs**

Plasmid Construct 244-26 = $(His)_6$-TEV-KRas (1-180) G12V
A pET22b(+) plasmid (Novagen) containing $(His)_6$-TEV-KRas (1-180) was used as a template for Quickchange Mutagenesis (Stratagene) to generate the G12V mutant version using the mutagenesis primer: 5'- GGTAGTTGGAGCTGTTGGCGTAGGCAAGAG-3' and the reverse complement. Resultant colonies were screened for the codon change by sequence.

Plasmid Construct 244-34 = $(His)_6$-TEV-Avi-tag-$(Gly)_8$-KRas (1-180)_G12V
An existing Avi-tTag-$(Gly)_8$-KRas (1-180)_G12V in pBlueBac4.5 (Thermo Fisher Scientific) construct was used as a template to add a 5' NdeI restriction site plus a $(His)_6$-TEV sequence (upstream of the Avi-tTag) and a 3' BamHI site (downstream of the stop codon) using overlapping primer PCR. The resulting PCR fragment was column purified, double-digested with NdeI + BamHI and cleaned up a second time prior to ligation. The fragment was ligated to a pET22b(+) vector (Novagen) double-digested with NdeI + BamHI and dephosphorylated with calf intestinal alkaline phosphatase (Roche) and transformed into chemically competent Top10F' cells. Resultant colonies were screened for the fragment insert and sequence confirmed.

***E. coli* plasmid Construct with BirA gene**
The *E. coli* B strain AVB101 (Avidity: Strain AVB101, an E. coli B strain (hsdR, lon11, sulA1), contains pBirAcm, an IPTG inducible plasmid containing the BirA gene engineered into pACYC184) was used as a source of pBirAcm plasmid. The plasmid was purified from a 5ml overnight culture grown in LB+10ug/ml Chloramphenicol. This plasmid was used for co-transformation with the plasmid carrying the Avi-tagged gene of interest.

Plasmid Construct 262-1 = GST-cRAF RBD (aa51-131)
A DNA fragment coding for amino acids 51-131 of c-Raf (RBD=Ras Binding Domain) with a 5' BamHI site and an EcoRI site at the 3' end was generated using PCR. The PCR fragment and the *E. coli* expression vector pGEX2T (GE Healthcare Life Sciences) were both double-digested with BamHI and EcoRI restriction enzymes and column purified to remove excess primers, nucleotides and enzymes. The two fragments were ligated together and transformed into chemically competent Top10 cells. Resultant colonies were screened for the fragment insert and sequence confirmed.

Baculovirus expression constructs

Plasmid Construct 244-6 (=BV1689) KRas FL, no tags, prenylated
Vector preparation: The insert of a previously made KRas construct, cloned into baculovirus transfer vector pBlueBac4.5/V5-$(His)_6$TOPO (Thermo Fisher Scientific, discontinued), was cut from the vector using NcoI (at the ATG site) and HindIII (downstream of the stop codon). The NcoI-HindIII vector fragment was agarose gel purified. Insert preparation: The full length KRas coding sequence (aa 1-188) with the activating mutation G12V was cut from a previously made construct using BspHI (at the ATG site, ligation compatible with NcoI) and HindIII (downstream of the stop codon). The KRas fragment was agarose gel purified. The prepared KRas (1-188) and pBlueBac4.5 vector fragments were ligated together and transformed into chemically competent Top10 cells (Thermo Fisher Scientific). Resultant colonies were screened for the fragment insert and sequence confirmed.

Plasmid Construct 244-35 (=BV3095) Avi-tag-$(Gly)_8$ –KRas G12V (2-188)
Using a full length construct (KRas FL (G12V) in pBB45 (Thermo-Fisher Scientific) as a template, a BamHI plus Avi-tag-$(Gly)_8$ sequence (Avidity©) was added to the 5' end and HindIII to the 3'end in one PCR reaction using overlapping primers. After gel purification of the PCR product, the PCR fragment was cut with BamHI and HindIII restriction enzymes, and ligated into pFastBac1 vector (Thermo Fisher Scientific) cut with the same enzymes and transformed into chemically competent Top10 cells (Thermo-Fisher). Resultant colonies were screened for the fragment insert and sequence confirmed.

Plasmid Constructs 151-18 bRaf FL (BV837) and 151-19 bRaf FL V600E (BV836)
The full length bRaf (wild type and V600E) coding sequence (amino-acids 1-765) was PCR amplified using previously obtained cDNAs as template. The PCR primers contained an XbaI site at the 5' end and a $(His)_6$-stop-SalI at the 3' end. The PCR fragment was gel purified and ligated into the XbaI and SalI sites of phosphatase-treated baculovirus transfer vector pBlueBac4.5 (Thermo Fisher Scientific). The ligation reaction was transformed into *E. coli* as above. Colonies were screened for insert and sequence verified.

Dual Plasmid Construct with MEK1 K97R-cAvi tag and BirA (BV2950)
A dual expression of BirA and N-terminal $(His)_6$-HRV3C-MEK1 K97R-$(Gly)_4$-C terminal Avi-tag (Avidity©) in the pFastBac Dual vector (Thermo Fisher Scientific) was generated. For convenient co-expression of biotin ligase with an AviTagged protein, the *E. coli* BirA gene was cloned into pFastBac Dual (Invitrogen) behind the p10 promoter. Using a 5' PCR primer containing a SmaI site and a 3' primer containing an XhoI site, the BirA coding sequence was amplified from the pBirAcm plasmid. The resulting PCR fragment was sequentially digested with SmaI and XhoI, spin column purified and ligated to pFastBac dual cut with the same enzymes. The AviTagged gene of interest was then cloned behind the Polyhedrin (PH) promoter. The human MEK1 K97R gene with a N-terminal $(His)_6$ followed by a HRV3C cleavage sequence and a C-terminal $(Gly)_4$-linker followed by an Avi-tag (Avidity©) was driven by the polyhedrin promoter.

***E. coli* protein expression**

Expression of plasmid Construct 244-34 = $(His)_6$-TEV-Avi-tag-$(Gly)_8$-KRas (1-180)_G12V
Plasmid 244-34 and a BirA (biotin ligase) plasmid were co-transformed into *E.coli* expression strain BL21 (DE3) (Stratagene/Agilent). The transformation was plated on dual LB plates with 100μg/mL carbenicillin and 20μg/mL of chloramphenicol. A single colony was used to grow an overnight starter culture in LB with 100μg/mL of carbenicillin and 20 μg of chloramphenicol at 37°C and this used to inoculate 2 x 1L cultures to a starting OD_{600} = 0.1 OD/mL. These pre-induction cultures were grown at 37°C, at 240rpm to an OD_{600} = 1.9 OD/mL. The temperature was turned down to 18°C and culture flasks were chilled without shaking and when chilled, D-biotin to 50 uM was added and then induced with IPTG to 0.5 mM. Flasks were grown overnight at 18°C at 240 rpm. Cells were harvested at OD_{600} = 3.9OD/mL, pelleted by centrifugation, supernatant poured off and the pellet stored at -80°C.

Expression of Plasmid Construct 244-26 = $(His)_6$-TEV-KRas (1-180) G12V
Plasmid 244-26 was transformed into *E. coli* expression strain Rosetta2 DE3 (EMD-Millipore) and plated for isolated colonies. A 300ml starter culture was grown in LB with 100μg/mL of carbenicillin and 20 μg/mL of chloramphenicol to an OD_{600}=0.95. The starter culture was used to inoculate a fermenter containing the following modified YEP medium (4% Casein Peptone, 2% Tastone 154, 1% Potassium Phosphate, (dibasic), 1% glycerin, 1% Glucose, 20μg/mL chloramphenicol and 50μg/mL carbenicillin) The culture was grown at 37°C to an OD_{600}=8-10. At this point the temperature was reduced to 18°C and

induced with 0.81mM IPTG and the culture continued to an approximate final OD_{600}=30. The cells were harvested and frozen at -80°C.

Expression of plasmid Construct 262-1 = GST-cRAF RBD (aa 51-131)
Plasmid 262-1 in the pGEX2T vector (GE Healthcare) containing a tac promoter, was transformed into *E. coli* BL21 expression cells (EMD-Millipore) and selected on LB plates containing 100µg/mL carbenicillin. A single colony was used to grow an overnight starter culture at 37°C in LB + carbenicillin. Used the starter culture to inoculate 4 x 1L cultures to a starting OD_{600} = 0.1 OD/mL. These pre-induction cultures were grown at 37°C, 240rpm to an OD_{600} = 0.7 OD/mL. The cultures were induced by adding IPTG to 0.5mM final concentration. The cultures were incubated with shaking at 37C for 4hrs. Cells were harvested by centrifugation. Pellets were stored at -80 °C until processed.

Insect cell protein expression

Preparation of Baculovirus for insect cell expression:
Baculovirus generation from transfer vector pBlueBac4.5 plasmids: The pBlueBac4.5 plasmids (Thermo Fisher Scientific) (KRas FL 244-6, bRaf 151-18 (BV737) and 151-19 (BV736)) were used to generate baculovirus by the co-transfection/plaque purification method [1]. Virus generated from the transfected insect cells was amplified using a standard low MOI infection method.

Bacmid and baculovirus generation: The KRas-Avi-tag 244-35 (BV3095) and MEK1 K97R-Avi-tag/BirA (BV2950) plasmid DNAs were used to generate bacmid DNA as instructed in the Bac-to-Bac manual (Invitrogen), in particular the section headed “Generating the Recombinant Bacmid”. The resultant plates of DH10Bac transformants contained a mix of small blue colonies and a few large white colonies. Two large white, well separated colonies were chosen for bacmid DNA preps. DNAs were prepared using the PowerPrep HP BAC Buffer Kit (OriGene, cat# NP10003/11448-100). The recombinant bacmid DNA was analyzed by PCR per the Bac-to-Bac manual, using a gene specific forward primer and the M13 Rev primer. Baculovirus expressing the recombinant proteins was generated using the Bac-To-Bac Expression System method (Invitrogen) following manufacturer’s protocol. The virus generated from the transfected insect *Sf9* cells was amplified using a standard low MOI infection method.

Expression of plasmid Construct 244-6 (=BV1689)—KRas FL, no tags, prenylated
Plasmid Constructs 151-18 bRaf FL (BV837) and 151-19 bRaf FL V600E (BV836)
To generate recombinant proteins, suspension cultures of insect cells (*Sf9, Sf21 or Tn5*) were seeded at a density of $1.5x10^6$cells/ml and infected with virus at an MOI of 10 or a with a 3% volume. The infected insect cells were cultured for 48 hours at 27°C shaking at 120 rpm using 2L glass Erlenmeyer flasks and serum free media. Cells were harvested two days post-infection, frozen and stored at -80°C.

Expression of Plasmid Construct 244-35 (=BV3095) Avi tag-$(Gly)_8$ –KRas G12V (aa 2-188)
To generate biotinylated Avi-tagged protein, a suspension culture of insect cells (*Tn5)* were seeded at a density of $1.5x10^6$ cells/ml and co-infected with virus at an MOI of 5:5 (BV3095 and BirA-expressing virus, BV1845, both at passage 2). D-biotin was added to 50µM final concentration. The infected insect cells were cultured for 48 hours at 27°C shaking at120 rpm using 2L glass Erlenmeyer flask and serum free media. Cells were harvested two days post-infection, frozen and stored at -80°C.

Expression of Dual Plasmid Construct with MEK1 K97R and Bir1 (BV2950)
To generate biotinylated MEK1 K97R, a suspension culture of insect cells (Sf9) were seeded at a density of $1.5x10^6$ cells/ml and infected with virus at an MOI of 10 or with a 3% volume. D-biotin was added to 50µM final concentration. The infected insect cells were cultured for 48 hours at 27°C at 120 rpm using

1L glass Erlenmeyer flasks and serum free media. Cells were harvested two days post-infection, frozen and stored at -80°C.

Protein purification

Cell lysis conditions

Large-scale expression cultures were grown using standard procedures, as described above. Cells were collected by centrifugation and resuspended in ~5x volumes of lysis buffers, as specified below. *E. coli* cells were lysed by sonication or Microfluidizer (Microfluidics) and insect cells were lysed by Dounce homogenization. Total cell lysates were centrifuged at 42,000 x g for 30 minutes at 4° C to obtain the soluble lysates.

Purification of KRas_EX244.26 (G12V, 1-180) expressed in *E. coli*

E. coli cells were lysed in IMAC buffer [25 mM Tris-HCl, pH 8.0; 250 mM NaCl; 5 mM MgCl2; 20 mM imidazole; 200 μM GDP; 5 mM 2-mecaptoethanol] with Benzonase endonuclease and protease inhibitors, and the soluble lysate was loaded onto a nickel-chelating Sepharose FF (GE Healthcare) IMAC column equilibrated with IMAC1 buffer A [25 mM Tris-HCl, pH 8.0; 250 mM NaCl; 5 mM MgCl2; 5 μM GDP; 5 mM 2-mercaptoethanol; 20 mM imidazole]. After the loading was completed, the column was washed with IMAC1 buffer A and then developed with a gradient to 100% (v/v) IMAC1 buffer B [25 mM Tris-HCl, pH 8.0; 250 mM NaCl; 5 mM MgCl2; 5 μM GDP; 5 mM 2-mercaptoethanol; 250 mM imidazole] over 20 column volumes. Fractions were analyzed by SDS-PAGE and pooled accordingly. 6xHis-tagged TEV protease (500 U/mg target protein) was added and the reaction mixture was then dialyzed overnight at 4° C against IMAC2 buffer A [25 mM Tris-HCl, pH 8.0; 150 mM NaCl; 2 mM MgCl2; 5 mM 2-mercaptoethanol] to remove the imidazole. After verifying by SDS-PAGE that digestion was completed the reaction mixture was loaded onto a second nickel-chelating column equilibrated with IMAC2 buffer A, which was then washed with IMAC2 buffer A and developed as above with a gradient to 100% (v/v) IMAC2 buffer B [25 mM Tris-HCl, pH 8.0; 150 mM NaCl; 2 mM MgCl2; 5 mM 2-mercaptoethanol; 250 mM imidazole] over 20 column volumes. Fractions were analyzed by SDS-PAGE and pooled accordingly; the TEV-cleaved KRas eluted in the IMAC2 flow-through, and could be aliquoted, frozen and stored at -80° C at this stage. This material was diluted 1/3x to 50 mM NaCl with Q dilution buffer [25 mM Tris-HCl, pH 8.0; 2 mM MgCl2; 1 mM TCEP] and loaded onto a Q HP (GE Healthcare) column equilibrated with Q buffer A [25 mM Tris-HCl, pH 8.0; 50 mM NaCl; 2 mM MgCl2; 1 mM TCEP], which was washed with Q buffer A and developed with a gradient to 100% (v/v) Q buffer B [25 mM Tris-HCl, pH 8.0; 1 M NaCl; 2 mM MgCl2; 1 mM TCEP] over 40 column volumes. Fractions were analyzed by SDS-PAGE and pooled accordingly; the highly-pure KRas eluted in the Q flow-through, ready for nucleotide loading and SEC as described below.

Purification of KRas_EX244.34 (N-Avi, G12V, 1-180) expressed in *E. coli*

E. coli cells expressing Kras (N-Avi) were lysed in IMAC1 buffer A [20 mM Tris-HCl, pH 8.0; 500 mM NaCl; 2 mM MgCl2; 5 μM GDP; 5 mM DTT; 5 mM imidazole] with Benzonase endonuclease and protease inhibitors, and the soluble lysate was loaded onto a HisTrap FF column equilibrated with IMAC buffer A. The column was washed with IMAC1 buffer A and developed with a gradient to 100% (v/v) IMAC1 buffer B [20 mM Tris-HCl, pH 8.0; 500 mM NaCl; 2 mM MgCl2; 5 μM GDP; 5 mM DTT; 500 mM imidazole] over 50 column volumes. Fractions were analyzed by SDS-PAGE, pooled accordingly, and treated with 6xHis-TEV protease (500 U/mg target protein) overnight at 4°C. Cleaved material was concentrated and buffer-exchanged on PD10 drip columns (GE Healthcare) equilibrated with IMAC2 Buffer A [20 mM Tris-HCl, pH 8.0; 500 mM NaCl; 2 mM MgCl2; 5 μM GDP; 5 mM DTT] . The buffer-exchanged material was loaded onto a HisTrap FF column equilibrated with IMAC2 buffer A and fractions were analyzed by SDS-PAGE and pooled accordingly; the cleaved protein is in the flow-through.

Purification of cRaf RBD (N-GST) expressed in *E. coli*
E. coli cells expressing cRAF RBD (N-GST) were lysed in GSTrap buffer A [PBS, pH 7.5; 5 mM DTT] containing Benzonase endonuclease and protease inhibitors, and the soluble lysate was loaded onto a GSTrap FF column (GE Healthcare) equilibrated with GSTrap buffer A. After washing the colum, cRAF RBD was eluted with GSTrap buffer B [50 mM Tris-HCl, pH 8.0; 150 mM NaCl; 10 mM reduced glutathione; 5 mM DTT]. The eluate was treated with thrombin (Roche, 1 U/mg target protein) overnight at 4° C, then further purified over a Superdex 75 size-exclusion column equilibrated with this same buffer.

Purification of KRas_BV1689 & BV3095 (G12V, full-length, prenylated, untagged & N-Avi) expressed in insect cells
Insect cells expressing full-length KRas were lysed in SP buffer A [25 mM Tris-HCl, pH 7.5; 5 mM NaCl; 2 mM MgCl2; 1 mM TCEP] with Benzonase endonuclease and protease inhibitors. The total lysate was centrifuged to obtain the soluble lysate, containing non-prenylated KRas. The cell pellet was washed twice with lysis buffer, resuspended in detergent-containing buffer [lysis buffer with 1% (w/v) CHAPS], mixed overnight at 4° C to extract the cell membrane-associated prenylated protein and centrifuged again to obtain the detergent-solubilized membrane extract. This was loaded onto an SP HP column equilibrated with SP1 buffer A [25 mM Tris-HCl, pH 7.5; 5 mM NaCl; 2 mM MgCl2; 1 mM TCEP; 1% (w/v) CHAPS]. The column was developed with a gradient to 100% SP1 buffer B [25 mM Tris-HCl, pH 7.5; 500 mM NaCl; 2 mM MgCl2; 1 mM TCEP; 1% (w/v) CHAPS] over 20 column volumes. Fractions were analyzed by SDS-PAGE and pooled accordingly, then dialyzed against SP2 buffer A [25 mM Tris-HCl, pH 7.5; 5 mM NaCl; 2 mM MgCl2; 1 mM TCEP; 1% (w/v) Na cholate] to exchange the detergent. This material was loaded onto a second SP HP column equilibrated with the same Na cholate-containing SP2 buffer A which was developed as above with a gradient to 100% (v/v) SP2 buffer B [25 mM Tris-HCl, pH 7.5; 500 mM NaCl; 2 mM MgCl2; 1 mM TCEP; 1% (w/v) Na cholate] over 20 column volumes. Fractions were analyzed by SDS-PAGE, and pooled accordingly for analysis by LC-MS (following extensive dialysis against non-detergent-containing buffer). The prenylated protein, a mixture of farnesyl- and geranylgeranyl-KRas, elutes in the flow-through or early in the salt gradient, followed by non-prenylated protein.

Purification of MEK1 (K97R, full-length, C-Avi) expressed in insect cells
Insect cells expressing MEK1 were lysed in IMAC1 buffer A [25 mM Tris-HCl, pH 7.5; 150 mM NaCl; 5% (v/v) glycerol; 5 mM imidazole; 1 mM TCEP] containing Benzonase endonuclease (EMD Millipore) and protease inhibitors (Roche cOmplete, EDTA-free). The soluble lysate was loaded onto a HisTrap FF IMAC column (GE Healthcare) equilibrated with IMAC1 buffer A, which was washed with 14% (v/v) IMAC1 buffer B [as above, but with 250 mM imidazole], then with 100% (v/v) IMAC1 buffer B to elute the heterogeneously phosphorylated recombinant MEK1 protein, which was treated with 6xHis-HRV 3C protease (20 U/mg target protein) and 6xHis-λ phosphatase (5 U/μg target protein) during dialysis against IMAC2 buffer A [25 mM Tris-HCl, pH 7.5; 150 mM NaCl; 5% (v/v) glycerol; 1 mM TCEP] overnight at 4° C. The resulting protein mixture was then passed over a second IMAC column equilibrated with IMAC2 buffer A and the flow-through material containing the MEK1 was recovered, concentrated, and passed over a Superdex 75 size-exclusion column (GE Healthcare). The progress of the purification was followed at all stages by SDS-PAGE. The final product was analyzed by LC-MS and determined to be non-phosphorylated and mono-biotinylated.

Purification of bRaf (wt- & V600E, full-length) expressed in insect cells
Insect cells expressing bRaf (wt- or V600E) were lysed in SP buffer A buffer [20 mM HEPES, pH 6.8; 50 mM NaCl; 10% (v/v) glycerol; 1 mM MgCl2; 1 μM ZnCl2; 5 mM DTT] containing Benzonase endonuclease and protease inhibitors. The soluble lysate was loaded onto a HiTrap SP HP column (GE Healthcare) equilibrated with the same buffer and the flow through was collected.

Loading of purified protein with defined nucleotide or nucleotide analog

To load the protein with nucleotide, a 100x molar excess of the nucleotide (*e.g.* GTPγS or GDP) was added to the protein followed by EDTA to 4 mM (2x the MgCl2 concentration). After incubation for 5 minutes at room temperature, MgCl2 was added to 8 mM (2x the EDTA concentration). The excess of nucleotide was removed by Superdex 75 size-exclusion chromatography in SEC buffer [25 mM Tris-HCl, pH 7.5; 50 mM NaCl; 2 mM MgCl2; 2 mM TCEP; and 1% (w/v) sodium cholate for prenylated proteins]. Fractions containing non-aggregated protein were pooled.

References

1. O'Reilly DR, Miller LK, Luckow VA. Baculovirus Expression Vectors: A Laboratory Manual. New York: Oxford University Press, Inc.; 1992.
