# Supplementary material for: Inhibition of prenylated KRAS in a lipid environment
Source: PLoS One. 2017 Apr 6;12(4):e0174706. doi: 10.1371/journal.pone.0174706 (PMC5383040; doi:10.1371/journal.pone.0174706)
Supplement: S2 Protocols — (PDF) [file pone.0174706.s002.pdf]

Inhibition of Prenylated KRAS in a Lipid Environment
Supporting Information: S2 Protocols

<u>Compound Synthesis and Characterization</u>

(2R,4S)-2-(4-chlorobenzyl)piperidin-4-amine
See reference [1]

5-bromo-N-((2R,4S)-2-(4-chlorobenzyl)piperidin-4-yl)-1H-indole-3-carboxamide: compound 2
To 5-bromo-1H-indole-3-carboxylic acid (125 mg, 0.521 mmol) was added NMP (6 ml), DIPEA (0.364 ml, 2.083 mmol) and (2R,4S)-2-(4-chlorobenzyl)piperidin-4-amine (124 mg, 0.552 mmol) . The mixture was stirred to fully dissolve, heat briefly as need, cool to room temperature, then add HATU (238 mg, 0.625 mmol). The reaction was stirred at room temperature for 2 hours, follow by LCMS. To the crude reaction was added 1 ml of DMSO, filter and purify by prep LC and lyophilized to give 115 mg of 5-bromo-N-((2R,4S)-2-(4-chlorobenzyl)piperidin-4-yl)-1H-indole-3-carboxamide as TFA salt in 39% yield. LCMS M+1 = 446.2/448.2, rt = 0.81 min. 1H NMR (<cd3od>) δ: 8.24 (d, J=2.0 Hz, 1H), 7.98 (s, 1H), 7.27-7.40 (m, 6H), 4.43 (t, J=4.5 Hz, 1H), 3.78 (dt, J=10.3, 3.5 Hz, 1H), 3.33-3.40 (m, 2H), 3.01 (qd, J=13.9, 7.2 Hz, 2H), 2.02-2.21 (m, 3H), 1.81-1.97 (m, 1H)

(2R,4S)-N-((1H-indol-3-yl)methyl)-2-(4-chlorobenzyl)piperidin-4-amine: compound 3
In a round bottom flask was added methanol (4mL), acetic acid (0.4 mL), methylene chloride (2 mL), 1H-indole-3-carbaldehyde and (2R,4S)-2-(4-chlorobenzyl)piperidin-4-amine the reaction was stir for 10 min. Then sodium cyano borohydride was added and stir at RT for 10 min., or until done by LCMS. The solvent was concentrated off, neutralize with saturated NaHCO3 solution, extract with ethyl acetate 2x, washed the organics with water, dried sodium sulfate, filtered and concentrated to residue. The residue was dissolved in DMSO, purified by prep LC and lyophilized. The product was free base by adding DCM washed with saturated sodium bicarbonate 2x, water 2x, saturated salt solution, dried with sodium sulfate, filtered and dried to constant mass to give119 mg of (2R,4S)-N-((1H-indol-3-yl)methyl)-2-(4-chlorobenzyl)piperidin-4-amine as the free base. LCMS M+1 = 354.1, rt = 0.64 min.

The HCl salt was made by adding methanol (1.50 ml) at room temperature then was added 1N HCl (aqueous, 0.70 ml) dropwise. The resulting solution was stirred at room temperature for 10 minutes. The reaction mixture was concentrated in vaccuo and then dissolved in ACN/water and lyophilized to give 115 mg of (2R,4S)-N-((1H-indol-3-yl)methyl)-2-(4-chlorobenzyl)piperidin-4-amine as HCl salt in 80% yield. LCMS M+1 = 354.2, rt = 0.63 min.**;** 1H NMR (DMSO-d6) δ: 11.40 (br s, 1H), 9.43 (br s, 2H), 9.02-9.24 (m, 2H), 7.68 (d, J=7.9 Hz, 1H), 7.62 (d, J=2.2 Hz, 1H), 7.40-7.46 (m, 3H), 7.28 (d, J=8.5 Hz, 2H), 7.16 (t, J=7.4 Hz, 1H), 7.05-7.12 (m, 1H), 4.18-4.42 (m, 2H), 3.99 (br s, 1H), 3.63 (br s, 1H), 3.45 (br d, J=17.3 Hz, 1H), 3.23 (br d, J=8.8 Hz, 1H), 3.07 (br dd, J=13.6, 5.0 Hz, 1H), 2.88 (br dd, J=13.4, 9.3 Hz, 1H), 2.15 (br s, 2H), 1.98-2.08 (m, 1H), 1.84-1.95 (m, 1H); 1H NMR (METHANOL-d4) δ: 7.70 (d, J=7.9 Hz, 1H), 7.56 (s, 1H), 7.47 (d, J=7.9 Hz, 1H), 7.38 (br d, J=8.5 Hz, 2H), 7.21-7.28 (m, 3H), 7.12-7.20 (m, 1H), 4.42-4.60 (m, 2H), 4.02 (br d, J=4.7 Hz, 1H), 3.79-3.89 (m, 1H), 3.41-3.56 (m, 2H), 2.97-3.13 (m, 2H), 2.36-2.49 (m, 1H), 2.19 (br dd, J=13.9, 4.4 Hz, 1H), 2.00-2.14 (m, 2H)

(S)-5-chloro-N-(1-((6-hydroxypyridin-3-yl)methyl)pyrrolidin-3-yl)-1H-indole-3-carboxamide: compound 4
To (S)-tert-butyl pyrrolidin-3-ylcarbamate (450 mg, 2.416 mmol) was added NMP (Volume: 12 mL) and 6-hydroxynicotinaldehyde (357 mg, 2.90 mmol). The reaction was stirred at room temperature for 45 minutes. Then sodium triacetoxyborohydride (1536 mg, 7.25 mmol) and stirred at room temperature for 16 hr followed by LCMS. To the crude was added 2 ml of water, filtered, purified by prep LC, the

desired fractions were combined, salt exchanged by adding HCl 6M aq (2.013 mL, 12.08 mmol) and lyophilized, to give 560 mg of (S)-tert-butyl (1-((6-hydroxypyridin-3-yl)methyl)pyrrolidin-3-yl)carbamate in 70% yield as HCL salt, used as is. LCMS M+1 = 294.4, rt = 0.41 min.

To (S)-tert-butyl (1-((6-hydroxypyridin-3-yl)methyl)pyrrolidin-3-yl)carbamate (560 mg, 1.698 mmol) was added HCl 4M in Dioxane (15 mL, 60.0 mmol), MeOH (Volume: 3 mL) and was stirred at room temperature for 2 hr followed by LCMS. The solvent was concentrated off to residue to give (S)-5-((3-aminopyrrolidin-1-yl)methyl)pyridin-2-ol as HCl salt used as is. Assume quantitative yield (1.698 mmol). LCMS M+1 = 194.2, rt = 0.12 min.

To 5-bromo-1H-indole-3-carboxylic acid (328 mg, 1.413 mmol) was added (S)-5-((3-aminopyrrolidin-1-yl)methyl)pyridin-2-ol (452 mg, 1.698 mmol), DCM (Volume: 12 mL, Ratio: 2.000), NMP (Volume: 6 mL, Ratio: 1.000) Huenig's Base (1.234 mL, 7.07 mmol), aza-HOBt (231 mg, 1.696 mmol) and then EDC (325 mg, 1.696 mmol). The reaction was stirred at room temperature for 1 hour then additional aza-HOBt (231 mg, 1.696 mmol) and EDC (325 mg, 1.696 mmol) were added. The reaction was allowed to stir overnight at room temperature for a total of 16 hours. The solvent (DCM) was concentrated off, 6 ml of DMF was added, filtered, purified by prep LC, and lyophilized. The product was re-dissolved in 1:1 ACN/water filtered and lyophilized again to give 354 mg of (S)-5-chloro-N-(1-((6-hydroxypyridin-3-yl)methyl)pyrrolidin-3-yl)-1H-indole-3-carboxamide as TFA salt in 51% yield. LCMS M+1 = 371.2, rt = 0.54 min.; ^{1}H NMR (<cd_3od>) δ: 8.09-8.15 (m, 1H), 7.95 (s, 1H), 7.70 (s, 2H), 7.40 (d, J=8.6 Hz, 1H), 7.17 (dd, J=8.6, 2.0 Hz, 1H), 6.60 (d, J=10.6 Hz, 1H), 4.57 (br. s., 1H), 4.19-4.35 (m, 2H), 3.76-4.03 (m, 1H), 3.63 (br. s., 1H), 3.39-3.57 (m, 1H), 2.64 (br. s., 1H), 2.27 (br. s., 1H)

References

1. Veenstra SJ, Hauser K, Schilling W, Betschart C, Ofner S. SAR of 2-benzyl-4-aminopiperidines NK1 antagonists. Part 21. synthesis of CGP 49823. Bioorganic & Medicinal Chemistry Letters. 1996;6(24):3029-34. doi: http://dx.doi.org/10.1016/S0960-894X(96)00563-X.
